# Therapist-guided online group forums in internet-based cognitive behavioral therapy for social anxiety disorder: A mixed-methods analysis

**DOI:** 10.1016/j.invent.2026.100936

**Published:** 2026-03-26

**Authors:** Alina Kneubühler, Anna K. Possehl, Ava Schulz, Thomas Berger

**Affiliations:** a Department of Clinical Psychology and Psychotherapy, University of Bern, Fabrikstrasse 8, 3012, Bern, Switzerland; b Independent Researcher

**Keywords:** Social anxiety disorder, ICBT, Therapist-guided online group, Discussion forum, Qualitative content analysis

## Abstract

Internet-based treatments for social anxiety disorder (SAD) have been widely studied in unguided and therapist-guided formats and, more recently, in group-guided formats that include guided online discussion forums. Little is known about what participants communicate and which processes unfold within these forum groups. This mixed-methods study analyzed communication in therapist-guided small-group forums integrated into an internet-based cognitive behavioral self-help program for SAD. Data stemmed from a three-arm randomized controlled trial and were limited to the forum arm.

A qualitative content analysis of 511 forum posts from 60 participants identified 18 main categories. Communication was dominated by therapist input, interactional group processes, symptom-related exchanges, and relational content, indicating that the forum functioned as a shared therapeutic space linked to the cognitive-behavioral intervention. Exploratory associations between category frequencies and treatment outcomes were examined for completers (*n* = 45). Higher frequencies of messages reflecting motivation (*r* = 0.41), alliance (*r* = 0.34), and observing positive consequences (*r* = 0.32) were associated with greater symptom reduction on the Social Phobia Scale, but not on the Social Interaction Anxiety Scale. Program adherence (completed modules) was significantly associated with 10 of 18 categories, primarily motivational/relational processes, responses to self-disclosure, difficulties, and evaluations of alternative behaviors.

Overall, therapist-guided online group forums extend the therapeutic learning environment by providing a social context in which motivational, relational, and treatment processes co-occur and are linked to outcome and adherence. Given the correlational and exploratory design, these associations should be interpreted cautiously and may represent mechanisms of change, markers of improvement, or both.

## Highlights

• First mixed-methods study of communication in guided group iCBT for SAD
• 2899 meaning units coded into 18 main categories
• Relational, motivational, and CBT processes co-occurred in discussions.
• Symptom reduction linked to motivational and alliance-related communication
• Guided forums may improve engagement and outcomes via social context.

## 1 Introduction

Social anxiety disorder (SAD) is among the most common mental illnesses, with a lifetime prevalence of approximately 4–15% (Kessler et al., 2005, Kessler et al., 2009; Stein et al., 2017). SAD is characterized by an intense fear of one or more social situations where one might be negatively evaluated or criticized (American Psychiatric Association, 2022). Consequently, affected individuals often avoid these situations or endure them with significant distress (Strauss and Mattke, 2018). Along with avoidance come emotions such as shame, guilt, and embarrassment, as well as physical symptoms such as blushing, stuttering, trembling, and sweating (Clark and Wells, 1995). Many individuals with SAD experience reduced functioning (Stein and Kean, 2000), lower quality of life (Dryman et al., 2016; Kessler, 2003), and significant social, personal, and professional impairments (Kessler, 2003; Stein and Kean, 2000). Without treatment, the disorder often has a chronic course and leads to comorbid conditions, social isolation, and significant impairments across various functional domains (Fehm et al., 2005; Lim et al., 2016; Stein and Kean, 2000). Despite the availability of various treatment methods, only 20–40% of individuals with SAD receive adequate care (Chapdelaine et al., 2018; Gross et al., 2005). Barriers range from systemic challenges and financial hurdles to illness-related factors, such as fear of judgment or stigmatization when seeking help (Goetter et al., 2020; Olfson, 2000).

### 1.1 Treatment methods for social anxiety disorder

The most common method for treating SAD is cognitive behavioral therapy (CBT). Its efficacy has been well-documented for SAD as well as other mental health disorders (Bandelow et al., 2021; Mayo-Wilson et al., 2014; Wolgensinger, 2015), with long-term effects that remain stable over time (Kindred et al., 2022). CBT is applied in both individual and group therapy contexts to treat people with SAD (Strauss and Mattke, 2018). Group therapy not only offers economic benefits but also fosters mechanisms that are less activated in individual therapy. Specifically, mutual support and sharing of experiences can be particularly beneficial for patients, as such interactions are often lacking due to disorder-specific behaviors such as social withdrawal and avoidance (Laurito et al., 2024; Strauss and Mattke, 2018).

Nevertheless, group therapy has demonstrated lower efficacy compared to individual therapy (Bandelow et al., 2021; Mayo-Wilson et al., 2014; Mörtberg et al., 2007). This discrepancy may stem from greater activation of disorder-specific patterns in group settings, leading patients to engage in heightened safety behaviors, increased avoidance, and reduced receptivity compared with individual therapy (Mörtberg et al., 2007; Stangier et al., 2003). To reduce the activation of disorder-related patterns in socially anxious individuals while preserving the benefits of group processes and addressing the treatment gap, online-based group therapies may offer a promising approach (Laurito et al., 2024).

The effectiveness of internet-based treatments has been well established, with SAD as one of the clinical fields in which research has been most extensive over the last few decades (Andersson and Berger, 2021), and meta-analyses reporting large between-group effect sizes (Kampmann et al., 2016; De Ponti et al., 2024). Most of the evidence comes from studies investigating guided internet-based cognitive-behavioral therapy (iCBT). Therein, participants progress through a structured self-help program based on CBT manuals and receive help and support from therapists via a secure email system. The advantages of internet-based treatments include their accessibility and efficient resource utilization (cost and therapist workload per patient) (Musiat and Tarrier, 2014). In addition, for individuals with SAD, internet-based treatment can lower the threshold for seeking help by reducing anxiety-provoking elements of face-to-face therapy. The online format can further support patients in conducting real-life exposure exercises by providing structured guidance for confronting feared situations (Biglbauer, 2024; Lee and Stapinski, 2012; Valkenburg and Peter, 2009).

Although many studies have explored the effectiveness of ICBT in individual settings, there is limited literature on group ICBT. One of the few studies addressing this is Schulz et al. (2016). Schulz et al. (2016) investigated the effectiveness of an ICBT self-help program combined with a guided online group forum, where participants could exchange experiences. Two experimental groups (one with individual therapist contact through the program and one with group forum interaction) were compared to a waitlist control group. Both experimental groups outperformed the control group in symptom reduction, but no significant differences in effectiveness were observed between the groups. Notably, the therapist's time spent in the group condition was reduced by 71%. More recent literature, such as Laurito et al.'s (2024) systematic review, highlights the potential of group ICBT. Findings regarding SAD indicate that internet-based interventions that incorporate elements of group therapy, specifically iCBT supplemented with therapist-moderated online discussion forums, are superior to passive control conditions and demonstrate efficacy comparable to individually guided internet-based interventions (Schulz et al., 2016; Titov et al., 2009).

### 1.2 Processes in therapist-guided online forums

The study by Schulz et al. (2016) identified no significant differences in overall effectiveness between the two experimental conditions (individually-guided vs. group-guided ICBT). However, differences emerged in the diary entries participants completed in the self-help program. Participants in the individual condition reported more instances of confronting anxiety-provoking situations and challenging negative thoughts compared to those in the group condition. Furthermore, the individual condition showed stronger positive correlations between symptom reduction and program adherence (e.g., number of modules completed and time spent in the program). However, the average number of modules completed did not differ significantly between the two experimental conditions. In the group condition, only the number of diary entries correlated significantly with symptom reduction (Schulz et al., 2016). Based on these findings, Schulz et al. (2016) hypothesized that distinct mechanisms underpinned the effectiveness of the two conditions. They proposed that group processes and exchanges in group forums compensated for the reduced exposure and practical application of the self-help program observed in the individual condition. This raises the question: What processes were associated with symptom reduction in the group condition?

To further examine the mechanisms underlying these findings, a quantitative secondary analysis of the same dataset was conducted, focusing on self-reported empowerment and working alliance (Duddeck et al., 2025). Results indicated that empowerment, as well as several dimensions of the working alliance (task/goal, bond, member-to-group, member-to-member), increased over the course of the program, with the group condition showing early increases in alliance. However, despite these overall improvements, no significant associations were found between member-to-member or member-to-group alliance and symptom reduction or adherence. In contrast, the task dimension of the working alliance was significantly associated with symptom reduction, whereas the bond dimension was significantly associated with adherence. Duddeck et al. (2025) suggested that this accelerated development of alliance may have been facilitated by group processes such as shared experiences and peer interactions.

Beyond these studies, limited research has focused exclusively on the content of online forums. Insights may nevertheless be drawn from studies on therapist–patient communication within individually guided ICBT. For example, Svartvatten et al. (2015) conducted a qualitative content analysis of therapist–patient email exchanges in an ICBT program for depression. They investigated the relationships between email content, symptom reduction, and treatment adherence. The authors identified significant correlations between symptom reduction and the categories of *alliance* (expression of emotional connection to the program/therapist) and *observes positive consequences* (resulting from trying alternative behaviors promoted by the program). Treatment adherence, operationalized as the number of completed modules, also correlated with these two categories as well as with *identifies patterns and problem behaviors*, *depressive thinking*, *tries alternative behavior*, *chooses alternative behavior* and *avoidance of treatment*. Soucy et al. (2018) partially replicated these findings in a study with a shorter ICBT program for depression and anxiety. They replicated the correlations between module completion and the categories of *tries alternative behavior* and *identifies patterns and problem behaviors*. Additionally, they found a positive correlation between symptom reduction and *observes positive consequences* and a negative correlation with *depressive thinking*.

The transfer of group processes from face-to-face to online settings is also discussed in clinical research. A broad range of group-therapeutic process factors has been identified, including instillation of hope, universality, imitative behavior, imparting of information, altruism, group cohesiveness, existential factors, self-disclosure, catharsis, interpersonal learning, development of socializing techniques, and the corrective recapitulation of the primary family group (MacKenzie, 1997; Yalom, 1996). Initial empirical findings suggest that traditional group processes can also emerge in online environments (e.g., Adamlje and Jendricko, 2020; Amulya, 2020; Ellett et al., 2022). For example, Berger (2011) conducted a qualitative content analysis of an unguided online forum for individuals with SAD, identifying mechanisms commonly found in face-to-face group therapies, such as universality, group cohesion, altruism, interpersonal learning, and self-disclosure. Other factors, particularly the development of socializing techniques and the corrective recapitulation of the primary family group, may be less likely to occur in online forums, potentially due to limited access to nonverbal communication cues (Johansson et al., 2013). Overall, existing studies provide initial indications that group-therapeutic processes can occur in online environments. However, empirical research remains limited, particularly regarding guided, asynchronous online group forums, and little is known about how these processes manifest in such settings or how they are associated with treatment outcomes.

### 1.3 Research gap and study aims

The investigation of change processes in digital interventions has been identified as a major research gap in the literature (Domhardt and Baumeister, 2021; Domhardt et al., 2025; Weinberg, 2020). A better understanding of these processes is essential for optimizing therapeutic programs and developing more targeted and efficient interventions. This is also relevant for SAD, as a substantial proportion of individuals continue to experience clinically significant symptoms following treatment, both in face-to-face and internet-based formats (Boettcher et al., 2013; McEvoy, 2007; McEvoy et al., 2012).

One promising yet understudied source of information on treatment-related processes is patient communication in therapist-guided online group forums embedded in iCBT programs. In the present study, these forums were conducted in small groups of participants in a shared discussion environment. Examining the content of these interactions may provide insights into therapeutically relevant processes and their potential associations with treatment outcome and adherence.

The present study, therefore, investigates the content discussed in a therapist-guided online forum for individuals with SAD and examines whether specific topics are associated with symptom change and treatment adherence. Given the limited research on the content of guided asynchronous online group forums, the study is exploratory, and no specific hypotheses were formulated.

## 2 Methods

In the present study, data from a previously published randomized controlled trial by Schulz et al. (2016) were used. A mixed-methods design was employed, integrating both qualitative and quantitative approaches.

### 2.1 Participants

Participants for the Schulz et al. (2016) study were recruited via the study website and through posts in online forums across Switzerland, Austria, and Germany. Participants were required to be at least 18 years old, possess self-assessed sufficient proficiency in German, and have access to both the internet and a computer. Exclusion criteria included ongoing psychological treatment, changes in medication during the last month prior to the study, or active suicidal plans. Further details about participant criteria can be found in Schulz et al. (2016) and the published study protocol (Schulz et al., 2014).

Out of 667 registrations, 204 individuals returned signed consent forms and were screened using the Social Phobia Scale (SPS; Mattick and Clarke, 1998; German version: Stangier et al., 1999) and Social Interaction Anxiety Scale (SIAS; Mattick and Clarke, 1998; German version: Stangier et al., 1999). Those exceeding predefined cut-offs (SPS > 22, SIAS >33) were invited to a telephone interview using the Structured Clinical Interview for DSM-IV – Axis I Disorders (SCID-I; First et al., 1995; German version: Wittchen et al., 1997) Participants meeting SAD diagnosis were randomized to one of three conditions: a waitlist control group that received access to the self-help program with individual therapist support after the 12-week primary endpoint; a guided individual iCBT condition with weekly therapist feedback; and a guided group iCBT condition in which the program was accompanied by participation in a therapist-guided online forum with six members. For this study, only data from the guided group ICBT condition (*n* = 60) were analyzed. This group had equal gender representation (30 men, 30 women), with a mean age of 35.89 years (SD: 11.31), and 48.3% presented with at least one comorbid condition.

### 2.2 Intervention

#### 2.2.1 Online program

The primary intervention was a self-help program for SAD comprising 8 modules (∼50 min per session), completed weekly, including exercises and diary entries. Table 1 provides a brief overview and explanation of the sessions' content. The program, based on the cognitive-behavioral model (Clark and Wells, 1995), covered psychoeducational content and strategies for modifying SAD-related cognitions and behaviors. The efficacy of this program has been shown in other studies (Berger et al., 2009, Berger et al., 2010, Berger et al., 2011; Boettcher et al., 2012; Stolz et al., 2018).

**Table 1:** Content of the internet-based self-help program for SAD.

Session	Content
Session 1: Motivational enhancement	Reasons to initiate change, definition of goals, recording of difficult social situations
Session 2: Psychoeducation	Information on SAD and its maintaining processes such as negative beliefs, self-focused attention and safety behaviors Development of an individual model for SAD
Session 3: Cognitive restructuring	Identification and modification of dysfunctional assumptions using a thought record
Session 4: Self-focused attention	Various exercises to reduce self-focused attention, e.g., short behavioral experiments
Session 5: Behavioral experiments	Planning and implementation of in vivo exposures
Session 6: Summary and repetition	Summary of the key elements of the treatment with emphasis on the importance of repeated practice (e.g., in vivo exposure)
Session 7: Healthy lifestyle and problem solving	Information on healthy lifestyle behavior (physical exercise and nutrition) Development of problem-solving skills
Session 8: Relapse prevention	Strategies for maintaining the skills learned, preparation for possible relapse

#### 2.2.2 Guided group forum

In addition to the self-help program, participants received support through a moderated group forum with six members per group who started treatment at the same time. Participants were consecutively allocated to the groups without randomization to minimize waiting times. Moderation was conducted by one psychotherapist-in-training and three master's students in psychology, who provided weekly feedback and encouraged the exchange of personal experiences, challenges, and questions. A licensed psychotherapist supervised the therapists. Participation in the forum was pseudonymous to ensure anonymity. Participants also had the opportunity to share their progress and journal entries with other group members.

### 2.3 Data

#### 2.3.1 Qualitative data

The 511 entries of the 10 moderated group forums from the study by Schulz et al. (2016) were used as text material. The entries consisted of messages from therapists (232, 45.4%) and group members (279, 54.6%).

#### 2.3.2 Quantitative data

##### 2.3.2.1 Social anxiety

The primary focus of the quantitative data analysis is the association of the content of the group forum and the SAD symptom reduction. *Social Phobia Scale* (SPS; Mattick and Clarke, 1998; German version: Stangier et al., 1999) and the *Social Interaction Anxiety Scale* (SIAS; Mattick and Clarke, 1998; German version: Stangier et al., 1999) were used. These tools assess two different domains of social anxiety symptoms: while the SIAS measures anxiety in social interaction situations (e.g., “I find it difficult to talk to other people”) (Mattick and Clarke, 1998), the SPS captures fear of negative evaluation in everyday situations (e.g., “I am afraid of blushing when I am with others”) (Šipka et al., 2023; Stangier et al., 1999). Both instruments have proven to be reliable measures for social anxiety in offline and online applications (Hedman et al., 2010; Stangier et al., 1999). Symptoms were assessed at baseline, at post-treatment (12 weeks; primary endpoint), and at a six-month follow-up. The present analyses were based on the baseline and post-treatment assessments.

##### 2.3.2.2 Adherence

In the present study, adherence is measured by the number of completed modules in the online CBT program. This operationalization has been employed in previous studies (Beintner et al., 2019; Soucy et al., 2018).

##### 2.3.2.3 Additional variables

In addition to the measures listed above, demographic and clinical data (e.g., comorbidities; therapeutic and medication history), as well as usage-related variables (e.g., number of messages posted, time spent in the forum, and forum clicks), were collected.

### 2.4 Data analysis

#### 2.4.1 Qualitative content analysis

Forum posts were analyzed with Mayring's (2015) qualitative content analysis method using the program MAXQDA+ (Version 24.3.0). A combination of deductive and inductive coding was applied. Deductive categories were based on existing literature on social anxiety (APA, 2013; Clark and Wells, 1995) as well as qualitative analyses of online forums (Berger, 2011) and therapist-patient interactions (Svartvatten et al., 2015). These categories were defined and refined before coding began. During the coding process, new inductive categories were identified from the material and added after discussion with the research partner.

The smallest unit of coding was a sentence. In line with comparable studies (e.g., Schneider et al., 2016), codes were assigned by message. If a category appeared multiple times within a message, the codes were combined to ensure each category was assigned only once. An exception was made for therapist messages, allowing a category to be assigned multiple times per message if the message addressed different individuals.

The coding procedure was conducted in several steps: Initially, one randomly selected forum was jointly coded by the two raters as a training exercise. During this step, the existing category system was refined, definitions were adjusted, and illustrative examples were compiled. Where necessary, new categories were inductively developed and integrated into the system. Subsequently, two additional forums were randomly selected and independently coded by each rater. Interrater reliability was assessed using MAXQDA+, with Cohen's Kappa (κ = 0.44) falling below the predefined threshold of 0.7, as recommended for qualitative content analysis (Mayring, 2015). Discrepancies were discussed, and the category system was revised accordingly. Following these adjustments, two further forums were randomly selected and again independently coded. Interrater reliability improved to a satisfactory level (κ = 0.72), and category saturation was reached, precluding the formation of additional categories. The initial forum was recoded, and the remaining five forums were randomly divided between the raters and coded independently. Any questions or ambiguities were discussed and resolved collaboratively. Upon completion of coding, the datasets were merged for analysis. To assess between-group variability in forum content, one-way analyses of variance (ANOVAs) were conducted across the ten groups for each main category based on group-level category frequencies.

#### 2.4.2 Quantitative analyses of group discussion content in relation to individual outcomes

To examine the relationships between discussion content, symptom reduction, and adherence, Spearman’s rank-order correlation coefficient (Spearman’s ρ) was used. Individual values from the participants on the measurement instruments (SPS, SIAS, Modules) were used and correlated with the number of categories assigned in the corresponding group. This analytic strategy was chosen because participants were exposed to the entire group discussion regardless of their own posting activity; thus, potential effects may arise not only from writing messages but also from reading and reflecting on other members' contributions. Such exposure can elicit group-therapeutic processes such as normalization and validation even in the absence of active participation (Harper and Cole, 2012). Consequently, the shared discussion environment, rather than individual posting behavior, was considered the relevant unit of analysis. The assumptions for Spearman's correlation were checked, and calculations were performed using SPSS (Version 29.0.2.0).

Symptom reduction was examined separately for SPS and SIAS and was calculated using residual gain scores. To do this, the difference between the z-standardized pre- and post-values was multiplied by the correlation between the two measurements (Z2 - Z1 * R12). According to Steketee and Chambless (1992), this method is appropriate for counteracting measurement errors in repeated measurements and controlling for initial differences between individuals. Adherence was operationalized as the number of completed modules and was derived from the program's user data.

A completers analysis was conducted for the calculation. Participants were classified as completers if post-intervention data were available for the outcome measures (SPS, SIAS). Baseline comparisons between completers and non-completers were conducted using independent-samples *t*-tests for continuous variables and χ^2^ tests for categorical variables, with effect sizes reported as Cohen's *d*. For 15 participants, no post-measurement data on symptoms were available, so only the data from *n* = 45 participants could be included.

## 3 Results

Participation varied considerably between individuals. On average, participants posted 4.83 messages (range: 0–40) and spent a mean of 164 min in the forum, indicating that while some participants were highly active, others contributed little or not at all. Engagement also differed between groups, with the total number of messages per forum ranging from 28 to 111. Detailed descriptive indicators of forum engagement at both the individual and group level are reported in Supplementary Table 1, while the distribution of content codes across the ten forums is shown in Supplementary Table 2.

### 3.1 Key themes discussed in group forums

A total of 2899 codes were identified. Duplicate messages were coded only once. Sections containing personal, non-disorder-related information (e.g., “I work as a software engineer”) or administrative instructions (e.g., “Could those of you who haven't yet completed the second questionnaire please do so now?”) were excluded from coding.

In total, 91 categories were created and subsequently grouped into 18 main categories. Table 2 provides an overview of the main categories, including descriptions, examples, as well as the total and average frequency per group. Across forums, communication was primarily characterized by therapist contributions (24.1%) and interactional group processes (20.7%). Therapist codes mainly comprised structuring, encouragement, normalization, and guidance of the discussion, whereas group processes reflected supportive exchanges, mutual feedback, and the sharing of personal experiences. A substantial proportion of the messages focused on the description of social anxiety symptoms and triggering situations (12.7%). Alliance/therapeutic relationship (9.3%) comprised expressions of appreciation toward the program, the therapists, or the group, as well as agreement with therapeutic tasks and goals. Response to self-disclosure (9.6%) captured active reactions to other members' posts, such as expressing understanding, asking questions, giving advice, or engaging in reciprocal self-disclosure. Emotional self-disclosure (4.0%) reflected the sharing of personal emotions in relation to social anxiety or to the implementation of the exercises. All remaining categories occurred at comparatively lower frequencies, including etiological factors (3.4%), difficulties in implementing treatment components (3.0%), tries alternative behavior (2.8%), observes positive consequences (2.1%), impact of disorder (2.0%), motivation (1.7%), rupture (1.2%), identifies patterns and problem behavior (0.9%), problems with techniques and administration (0.8%), chooses alternative behavior (0.6%), observes negative consequences (0.6%), and comorbidity (0.5%) (see Table 2).

**Table 2:** List of deductive (D) and inductive (I) main categories ordered by frequency.

Main categories	Number of categorized comments (%)	Per category m (SD)	Definition	Example
Therapist codes (I)	698 (24.1%)	69.8 (24.5)	Texts in which the therapist responds to a person's messages, structures them or encourages the writing of messages, e.g., through reinforcement, encouragement, normalization, guidance, etc.	Do you have any other tips for us that you use to visualize your successes? It would be great if you could share your experiences with us here. (VP 20)
Group processes (D)	599 (20.7%)	59.9 (48.8)	Texts in which group factors can be observed, including supportivity, self-revelation, learning and psychological work	I find it helpful to see that you all have similar fears and that we know how the others feel without much explanation. (VP 74)
Symptoms (D)	368 (12.7%)	36.8 (32.2)	Texts describing cognitive, emotional, physical, or behavioral symptoms of social anxiety or triggering situations.	In meetings, for example, I often don't think primarily about what I'm doing, but rather about how I'm coming across. Am I sitting correctly, do I dare to look at someone? […] How do I have to present myself, what appearance do I have to convey to “appear” as good as possible? (VP 97)
Response to self-disclosure (D)	278 (9.6%)	27.8 (19.4)	Texts in which a person reacts to another person's self-disclosure, e.g., by acknowledging it, asking questions, expressing understanding, giving tips or reacting with self-opening, etc.	I completely understand you and I feel the same way. (VP 166)
Alliance/Therapeutic relationship (D)	270 (9.3%)	27 (24.7)	Texts in which a person expresses a positive relationship to the tasks or goals of the program, to the therapist, to individual group members, to the entire group or to the program	For the first time I have found here an enormously to the point help/description on social anxiety - I really have to thank you for that, since this online help is provided free of charge! THANK YOU THANK YOU THANK YOU - great!!! (VP 81)
Emotional self-disclosure (D)	116 (4.0%)	11.6 (11.5)	Texts in which a person expresses their emotions, e.g., in relation to everyday situations, exercises in the program, etc.	In addition to my social anxiety, I often feel very down and depressed for days at a time. My nerves are then correspondingly thin, I find it hard to concentrate, find it hard to play with my son and of course the anxieties then have more fertile ground to spread (VP 138)
Etiological factors (I)	100 (3.4%)	10 (3.7)	Texts speculating on factors contributing to the development of social anxiety, such as past experiences or traits.	I can also remember a formative experience in 5th grade: we read a story in German class in which a woman drinks from a fountain of youth and becomes a baby again. […] The teacher asked: “What happened?” and I stood up and said: “The woman had a baby”. The whole class laughed uproariously. From then on, I never dared to say anything unprompted and panicked about being approached by the teacher and having to give an answer to something. […] (VP 186)
Difficulties with the program (I)	87 (3.0%)	8.7 (6.8)	Texts in which a person expresses difficulties or obstacles in coping with social anxiety or implementing the program	It's not easy to face your fears, especially when you've been carrying them around with you for over 30 years. A negative automatism has become ingrained. (VP 81)
Tries alternative behavior (D)	80 (2.8%)	8.0 (6.3)	Texts in which a person successfully performs or at least attempts to perform alternative behaviors or exercises	Questioning my fears in particular gives me a lot of strength and security. In situations where I am afraid, I think about it and think about why. I have never dealt with that. (VP 122)
Observes positive consequences (D)	62 (2.1%)	6.2 (5.4)	Texts in which a person experiences positive change as a result of an alternative behavior or exercise in the program	I grab a long, thick grass stalk and walk home with it in my mouth. Let's see how people react. I notice that not even half of the passers-by notice. […] Either way, I find it pleasant: if my stupid stalk is not noticed, there is no risk of devaluation. Others smile or laugh. Well, no offense, I'm making them happy. Maybe one or two of them will also devalue me and think to themselves “What an idiot”. I don't notice, so it doesn't hurt. Well, I can dare to relax and do whatever I feel like without (painful) consequences! (VP 97)
Impact of disorder (I)	59 (2.0%)	5.9 (6.1)	Texts in which emotional, social, physical, or professional consequences of the SAD are expressed	I quit my studies because I was too afraid of public speaking. (VP 133)
Motivation (I)	50 (1.7%)	5.0 (4.0)	Texts in which a person expresses their motivation for behavior change or program participation	Something must change. So, I'm already looking forward to what lies ahead. (VP 195)
Rupture (D)	34 (1.2%)	3.4 (3.7)	Texts in which tensions and problems between a person and goals, tasks, therapist, group members, the entire group and/or the online program become visible or in which a person refuses or discontinues treatment	I'm sitting alone on the train and thinking about whether I want to change at all. I have shared my “birthday invitation” protocol entry. If I go to this event at all, it will be to apply my findings from this study. I don't like these celebrations and honestly don't know why I should like them. (VP 218)
Identifies patterns and problem behavior (D)	27 (0.9%)	2.7 (2.6)	Texts in which a person identifies a connection between internal or external behavior and the effect on their own affective state	I have also noticed that I am sometimes too preoccupied with my fears and thoughts and therefore have no resources to focus my attention on the world around me. (VP 36)
Problems with techniques and administration (D)	22 (0.8%)	2.2 (1.8)	Texts in which a person expresses problems with the technology and/or organization of the program	Hi, I'm having a bit of a technical problem editing the anxiety hierarchies - instead of the editing window opening, I'm getting a Yahoo page. Can anyone help me with this? (VP 114)
Chooses alternative behavior (D)	17 (0.6%)	1.7 (1.7)	Texts in which a person expresses thoughts of using alternative behaviors or exercises in the future, or actively makes plans for implementing alternative behaviors or exercises	I will try to concentrate more closely on my thought processes over the next few days when I find myself in anxiety situations. (VP 138)
Observes negative consequences (I)	17 (0.6%)	1.7 (1.6)	Texts in which a person describes that no improvement has occurred despite the change in behavior or even negative consequences have been experienced as a result of the change in behavior	Today I had another very negative experience. I had to go to an owners' meeting. I tried to stay relaxed from the start and prepared myself to some extent. […] In the beginning, I also tried to actively participate in the conversation and simply ignored or suppressed my fear at first. But instead of becoming more relaxed at some point, the tension continued to rise until I didn't say much during the conversations. I had the feeling that everyone was looking at me and at some point, I closed up inside. (VP 191)
Comorbidity (I)	15 (0.5%)	1.5 (1.7)	Texts in which other comorbid disorders or symptoms are reported that preceded, occurred simultaneously with or developed from the SAD	Over the past three years, I've developed panic attacks, e.g., while driving on highways or being in crowded spaces like concerts or lectures. (VP 150)

To examine whether content patterns differed between groups, between-group variability was assessed across the ten groups. No significant differences were observed for most main categories. However, a significant between-group difference was found for the category *therapist codes* (*F*(1, 8) = 8.35, *p* = .020).

### 3.2 Correlations between main categories, symptom reduction, and adherence

Table 3 describes the associations between the main categories and symptom reduction. In line with the analytic strategy outlined in the Method section, all correlational analyses were conducted using a completers sample (*n* = 45), that is, including only participants with available post-treatment data on the SPS and SIAS. Baseline comparisons between completers and non-completers revealed a significant difference in SPS scores (*t*(56) = −2.07, *p* = .043, *d* = −0.65), with completers showing lower baseline SPS scores than non-completers. No differences were observed for SIAS (*t*(56) = −0.41, *p* = .681), age (*t*(15.15) = 0.06, *p* = .955), gender (*χ*^2^(1) = 3.57, *p* = .058), or comorbidity (*χ*^2^(1) = 0.03, *p* = .871).

**Table 3:** Correlations between main categories, symptom reduction and adherence.

	Residual gain scores SPS Spearman's r	Residual gain scores SIAS Spearman's r	Modules completed Spearman's r
Residual gain scores SPS	1.000	0.799⁎⁎	0.036
Residual gain scores SIAS	0.799⁎⁎	1.000	−0.041
Therapist codes	0.093	0.112	0.071
Group processes	0.281	0.160	0.329⁎
Symptoms	−0.180	−0.137	−0.074
Response to self-disclosure	0.114	0.065	0.383⁎⁎
Alliance/therapeutic relationship	0.340⁎	0.242	0.314⁎
Emotional self-disclosure	0.029	0.047	0.245
Etiological factors	0.216	0.089	0.110
Difficulties in program	0.212	0.067	0.371⁎
Tries alternative behavior	0.282	0.212	0.316⁎
Observes positive consequences	0.322⁎	0.201	0.313⁎
Impact of disorder	−0.123	−0.077	0.032
Motivation	0.409⁎⁎	0.258	0.299⁎
Rupture	0.096	0.065	0.276
Identifies patterns and problem behavior	0.099	0.066	0.549⁎⁎
Problems with technique and administration	−0.130	−0.133	−0.046
Chooses alternative behavior	0.128	−0.039	0.381⁎⁎
Observes negative consequences	0.239	0.120	0.393⁎⁎
Comorbidity	−0.285	−0.150	−0.252
Forum entries (per person)	−0.036	−0.001	0.499⁎⁎
Time spent in forum (per person)	0.087	0.020	0.203
Clicks in forum (per person)	0.045	−0.044	0.295⁎

Note. SPS = Social Phobia Scale, SIAS = Social Interaction Anxiety Scale. The residual change scores were calculated using the formula Z_2_ − (Z_1_ ∗ R_12_). A positive correlation indicates that the number of a category is associated with symptom deterioration, while a negative correlation indicates that it is linked to symptom improvement.

⁎ *p* < .05.

⁎⁎ *p* < .01.

Significant associations with symptom change emerged for the SPS but not for the SIAS. Residual gain scores on the SPS were significantly correlated with the categories alliance/therapeutic relationship (*r* = −0.34, *p* < .05), observing positive consequences (*r* = −0.32, *p* < .05), an motivation (*r* = −0.41, *p* < .01). No other categories were significantly associated with SPS residual gain scores (all *ps* > 0.05), and none of the correlations with SIAS residual gain scores reached statistical significance (all *ps* > 0.05). Adherence, operationalized as the number of completed modules, was significantly associated with several categories (see Table 3).

In addition, the number of completed modules was significantly correlated with the number of forum entries per participant (*r* = 0.50, *p* < .01) and the number of clicks in the forum (*r* = 0.30, *p* < .05), whereas time spent in the forum was not significantly associated with adherence (*r* = 0.20, *p* > .05).

## 4 Discussion

The present study examined the content of therapist-guided online group forums embedded in an internet-based CBT program for social anxiety disorder and explored how specific interaction patterns were associated with symptom change and treatment adherence.

### 4.1 Themes discussed in group forums

Across forums, communication was dominated by therapist input, interactional group processes, symptom-related exchanges, and relational content. This profile resembles central process factors known from face-to-face group psychotherapy, such as universality, mutual support, and interpersonal learning (Yalom, 1996; MacKenzie, 1997), and aligns with previous qualitative findings from unguided online forums for social anxiety (Berger, 2011). The relatively high proportion of responses to self-disclosure and alliance-related statements indicates that the forum was not merely used for information exchange but functioned as a shared therapeutic space in which group cohesion and a sense of common purpose could develop, even in an asynchronous text-based format (Adamlje and Jendricko, 2020; Ellett et al., 2022).

At the same time, many posts focused on the description of symptoms and disorder-related experiences. For individuals with social anxiety, who typically avoid self-disclosure and fear negative evaluation, such communication may constitute a corrective interpersonal experience (Clark and Wells, 1995; Sparrevohn and Rapee, 2009). The anonymous and structured setting likely facilitated normalization and reduced perceived social threat, thereby lowering barriers to engaging with therapeutic tasks.

In addition, the presence of behavior change–related categories (e.g., trying alternative behaviors, identifying patterns and problem behaviors, observing positive consequences) shows that the forum was closely intertwined with the CBT intervention itself. Rather than serving as a purely supportive add-on, it extended the therapeutic learning environment by providing opportunities for feedback, reinforcement, and vicarious learning, which are processes central to cognitive-behavioral interventions (Bandura, 1977; Davis et al., 2017).

### 4.2 Associations between categories, symptom reduction, and adherence

Three categories, motivation, alliance/therapeutic relationship, and observing positive consequences, were associated with symptom reduction on the SPS. Importantly, these associations do not imply that discussing these topics caused symptom change. It is equally plausible that participants who experienced greater improvement were more likely to express motivation, relational closeness, or positive treatment effects in the forum, or that third variables influenced both.

Motivation showed the strongest association with symptom improvement. From a self-determination theory perspective (Ryan and Deci, 2000), the expression of motivation may reflect autonomous engagement with treatment goals, which has been linked to better outcomes in internet interventions (Donkin and Glozier, 2012). In a group context, motivational statements may additionally contribute to a shared motivational climate that enhances persistence in exposure exercises and cognitive restructuring.

The association between alliance/therapeutic relationship and symptom change extends previous findings on the therapeutic alliance in internet interventions (Berger, 2017, Berger, 2025; Probst et al., 2019) to a guided online group format. Importantly, the coded content captured the alliance not only to the therapist but also to the program and the group. This supports recent conceptualizations of multiple alliance targets in digital treatments and suggests that a positive relationship to the treatment context as a whole may be associated with clinical improvement (Gómez Penedo et al., 2020; Berger, 2025).

Observing positive consequences was also related to symptom reduction. In line with learning theory, communicating successful behavioral experiments may function as direct and vicarious reinforcement (Bandura, 1977). Similar associations have been reported in content analyses of therapist–patient communication in individually guided ICBT (Svartvatten et al., 2015; Soucy et al., 2018), suggesting that this process may represent a transdiagnostic mechanism across digital formats. The forum may amplify these effects by making behavioral gains visible to the entire group, thereby increasing outcome expectancies and self-efficacy.

No statistically significant associations were found for the SIAS. This difference may reflect differences in construct, with performance-related fears of negative evaluation, as assessed by the SPS, and anxiety in social interactions, as measured by the SIAS (Mattick and Clarke, 1998). The structured and task-focused nature of the forum, which emphasized activities such as exposure exercises and cognitive restructuring, may have been more closely aligned with performance-related fears than with anxiety in spontaneous interpersonal encounters. In addition, selective attrition, with completers showing lower baseline SPS scores, may have contributed to the SPS-related findings and should be considered when interpreting the results.

Adherence was associated with a broader range of categories, particularly those reflecting active cognitive-behavioral work (e.g., identifying patterns and problem behavior, as well as trying or planning alternative behaviors), as well as relational and interactional processes. In addition, the number of forum entries per participant showed a substantial correlation with the number of completed modules, suggesting that active participation in the forum and engagement with the program tended to co-occur. This may partly reflect a general engagement disposition; at the same time, it is also possible that the forum functioned as a social context that helped participants remain involved in the intervention, in line with models of supportive accountability in digital treatments (Mohr et al., 2011).

Taken together, the findings suggest that therapist-guided online group forums embedded in ICBT can constitute a multifaceted therapeutic context in which disorder-related self-reflection, interpersonal exchange, and active cognitive-behavioral work co-occur. The observed associations indicate that both relational processes and task-focused engagement are linked to treatment adherence and, for performance-related social anxiety, to symptom improvement. Rather than serving as a purely supportive adjunct, the forum appears to extend the therapeutic learning environment by integrating social and behavioral mechanisms central to both group psychotherapy and digital interventions.

### 4.3 Strengths and limitations

This study has several strengths. It is among the first to systematically examine the content of therapist-guided online group forums for individuals with social anxiety disorder embedded in an ICBT program, thereby addressing a clear research gap regarding the processes occurring in this format. By drawing on data from a well-conducted randomized controlled trial with a clinical sample, the study benefits from high ecological validity and a clearly defined treatment context. The relatively large corpus of forum material and the acceptable interrater reliability further strengthen the robustness of the qualitative findings. In addition, the mixed-methods design, combining qualitative content analysis with quantitative associations between communication patterns, symptom change, and adherence, allows a more comprehensive understanding of how group-related and cognitive-behavioral processes may be linked to treatment engagement and outcome.

However, several limitations should be considered when interpreting the findings. The correlational nature of the results underlines the need for cautious interpretation. Future studies using experimental designs are required to disentangle whether the identified communication patterns act as mechanisms of change, markers of improvement, or both, and to determine how online group formats can be optimized to foster these processes. Reliance on a completers sample is another limitation. Although baseline comparisons indicated largely comparable characteristics between completers and non-completers, excluding participants with missing post-treatment data may have reduced generalizability and introduced bias, particularly given the lower baseline SPS scores among completers. In addition, the analytic strategy combined individual-level symptom data with group-level category frequencies. Because participants within the same forum shared identical content values, the effective sample size was determined by the number of groups rather than the number of individuals. This lack of independence among observations, together with the small number of groups and variability in therapist contributions, limits statistical power and requires cautious interpretation of the reported associations. Furthermore, to increase sensitivity in a largely underexplored field, correlations were tested using an alpha level of *p* < .05. While this approach allowed the detection of smaller effects, it also increased the risk of Type I errors, and the findings should therefore be regarded as preliminary and in need of replication in larger samples with independent observations and more conservative statistical thresholds. Relatedly, adherence was operationalized as the number of completed modules. Although this is a widely used indicator in research on digital interventions, it captures only one aspect of engagement. Future studies could complement this measure with additional usage indicators, such as time spent in the program or the number of diary entries, to obtain a more differentiated understanding of treatment involvement (Beintner et al., 2019; Christensen et al., 2009). Finally, the data were collected several years ago. Since then, norms of online communication, platform characteristics, and users' familiarity with digital interaction formats have changed considerably. These developments may influence both the quantity and the quality of online self-disclosure and interaction patterns and, therefore, limit the direct transferability of the findings to contemporary guided online group forums.

### 4.4 Implications for future research and practice

Future research should move beyond purely correlational approaches and investigate how forum processes, treatment engagement, and symptom change are temporally and causally related. In particular, it will be important to determine what precedes what: whether changes in communication are followed by symptom improvement, or whether participants report more motivation, self-disclosure, and treatment gains because they are already improving. Longitudinal and microanalytic designs that capture the sequence of therapist and participant contributions over time would be especially valuable for clarifying this directionality and for identifying communication patterns that may serve as potential mechanisms of change.

From a clinical perspective, the findings suggest that guided online group forums can be more than a supportive add-on, providing a social context in which motivational, relational, and cognitive-behavioral processes co-occur. Structuring forums to actively facilitate mutual feedback, the sharing of behavioral successes, and a positive working relationship with the treatment context may therefore enhance both adherence and clinical outcomes. For individuals with a pronounced fear of negative evaluation, such formats may lower the threshold for engaging in exposure-related tasks and the intervention as a whole, thereby supporting the uptake of evidence-based treatments. These insights are particularly relevant for optimizing digital interventions, as they point to process variables that could be deliberately fostered through therapist behavior and forum design.

## 5 Conclusion

This study provides one of the first systematic insights into what is discussed in therapist-guided online group forums embedded in an ICBT program for social anxiety disorder and how these communication patterns are associated with treatment engagement and outcome. Across forums, communication was characterized by a combination of relational, motivational, and task-focused content, suggesting that the forums functioned as integral parts of the therapeutic environment rather than purely supportive adjuncts.

Higher frequencies of messages reflecting motivation, alliance/therapeutic relationship, and the observation of positive consequences were associated with greater reductions in performance-related social anxiety, whereas adherence was linked to a broader range of interactional and cognitive-behavioral processes. These patterns suggest that forum communication may serve as an indicator of how participants engage with the intervention and whether therapeutic change is occurring.

At the same time, the correlational and exploratory nature of the analyses precludes causal conclusions. The identified communication patterns may represent mechanisms of change, markers of improvement, or both, but they point to clinically relevant processes that could be deliberately facilitated through therapist behavior and forum design.

Taken together, guided online group forums appear to provide a social learning context in which interpersonal exchange, motivational processes, and active cognitive-behavioral work co-occur and are jointly linked to treatment adherence and symptom improvement. A better understanding of these processes may help to optimize digital interventions for social anxiety disorder and to design scalable treatment formats that combine the efficiency of ICBT with the therapeutic potential of group interaction.

## Declaration of competing interest

The authors declare the following financial interests/personal relationships which may be considered as potential competing interests: Given his role as member of the editorial board, co-author Prof. Dr. Thomas Berger had no involvement in the peer review of this article and had no access to information regarding its peer review. Full responsibility for the editorial process for this article was delegated to another journal editor. If there are other authors, they declare that they have no known competing financial interests or personal relationships that could have appeared to influence the work reported in this paper.

## Appendix A Supplementary data

Supplementary tables Image 1
