# Supplementary material for: Therapist-guided online group forums in internet-based cognitive behavioral therapy for social anxiety disorder: A mixed-methods analysis
Source: Internet Interv. 2026 Mar 26;44:100936. doi: 10.1016/j.invent.2026.100936 (PMC13068860; doi:10.1016/j.invent.2026.100936)
Supplement: Supplementary file 1 — Supplementary tables [file mmc1.docx]

**Supplementary Table 1**

*Descriptive data on group characteristics and forum engagement*

| Group | 1 | 2 | 3 | 4 | 5 | 6 | 7 | 8 | 9 | 10 | Total |
| --- | --- | --- | --- | --- | --- | --- | --- | --- | --- | --- | --- |
| Participants | 6 | 6 | 6 | 6 | 6 | 6 | 6 | 6 | 6 | 6 | 6 |
| Study Dropouts | 1 | 3 | 1 | 1 | 2 | 1 | 2 | 2 | 2 | 0 | 15 |
| Gender distribution (proportion of female participants) | 0.500 | 0.333 | 0.333 | 0.833 | 0.333 | 0.667 | 0.600 | 0.833 | 0.400 | 0.167 | 0.500 |
| Mean age | 30.3 | 39.5 | 39.8 | 31.5 | 34.8 | 32.0 | 32.0 | 38.7 | 38.4 | 41.7 | 35.9 |
| Mean SPS pre | 35.2 | 43.3 | 39.2 | 39.8 | 39.3 | 41.5 | 40.0 | 40.8 | 36.8 | 33.0 | 38.9 |
| Mean SIAS pre | 53.3 | 53.0 | 41.0 | 49.7 | 44.0 | 54.0 | 55.6 | 57.2 | 48.0 | 56.2 | 51.2 |
| Mean modules completed | 7.7 | 6.0 | 6.3 | 8.0 | 5.5 | 7.2 | 3.8 | 6.3 | 5.2 | 8.0 | 6.5 |
| Messages total | 32 | 28 | 35 | 79 | 49 | 111 | 36 | 32 | 40 | 69 | 511 |
| Messages participants | 14 | 13 | 21 | 51 | 24 | 74 | 13 | 15 | 21 | 33 | 279 |
| Messages therapist | 18 | 15 | 14 | 28 | 25 | 37 | 23 | 17 | 19 | 36 | 232 |
| Minimum messages per participant | 0 | 5 | 1 | 3 | 1 | 6 | 1 | 1 | 5 | 2 | 0 |
| Maximum messages per participant | 5 | 6 | 8 | 12 | 5 | 40 | 2 | 5 | 8 | 9 | 40 |
| Mean messages per participant | 2.2 | 2.2 | 3.5 | 3.5 | 4.0 | 12.8 | 4.0 | 2.7 | 3.9 | 5.3 | 4.8 |
| Mean clicks in forum (per participant) | 75.1 | 37.5 | 69.2 | 187.3 | 69.7 | 226.0 | 93.3 | 58.5 | 80.4 | 124.7 | 100.8 |
| Mean time spent in forum (seconds per participant) | 7281 | 3198 | 5318 | 2032 | 5830 | 18814 | 9814 | 4796 | 11236 | 12460 | 9840 |

*Note.* Calculations for groups 7 and 9 were made with five participants each, because two participants retracted their data from the study.

**Supplementary Table 2**

*Overview of the frequencies of the main categories across the ten group forums*

| Group | 1 | 2 | 3 | 4 | 5 | 6 | 7 | 8 | 9 | 10 | Total |
| --- | --- | --- | --- | --- | --- | --- | --- | --- | --- | --- | --- |
| Codes total | 145 | 164 | 202 | 427 | 306 | 724 | 154 | 197 | 247 | 333 | 2899 |
| Therapist codes | 46 | 45 | 39 | 64 | 84 | 102 | 70 | 59 | 76 | 113 | 698 |
| Group processes | 20 | 31 | 47 | 108 | 70 | 176 | 15 | 37 | 40 | 55 | 599 |
| Symptoms | 18 | 19 | 10 | 82 | 28 | 106 | 28 | 32 | 39 | 6 | 368 |
| Response to self-disclosure | 15 | 19 | 20 | 50 | 18 | 73 | 10 | 20 | 20 | 33 | 278 |
| Alliance / Therapeutic relationship | 6 | 17 | 25 | 32 | 34 | 90 | 3 | 14 | 16 | 33 | 270 |
| Emotional self-disclosure | 4 | 7 | 6 | 19 | 7 | 42 | 4 | 9 | 10 | 8 | 116 |
| Etiological factors | 10 | 4 | 10 | 11 | 12 | 9 | 10 | 5 | 12 | 17 | 100 |
| Difficulties in program | 6 | 3 | 10 | 13 | 7 | 24 | 4 | 2 | 4 | 14 | 87 |
| Tries alternative behavior | 2 | 7 | 6 | 9 | 13 | 21 | 0 | 2 | 8 | 12 | 80 |
| Observes positive consequences | 1 | 4 | 6 | 7 | 13 | 14 | 0 | 0 | 5 | 12 | 62 |
| Impact of disorder | 3 | 2 | 1 | 10 | 4 | 21 | 3 | 9 | 5 | 1 | 59 |
| Motivation | 3 | 0 | 11 | 5 | 6 | 6 | 0 | 3 | 4 | 12 | 50 |
| Rupture | 2 | 2 | 5 | 1 | 1 | 11 | 1 | 0 | 2 | 9 | 34 |
| Identifies patterns and problem behavior | 4 | 1 | 1 | 4 | 2 | 9 | 0 | 2 | 1 | 3 | 27 |
| Problems with technique and administration | 2 | 0 | 0 | 4 | 3 | 5 | 4 | 2 | 1 | 1 | 22 |
| Chooses alternative behavior | 1 | 1 | 3 | 5 | 1 | 4 | 0 | 0 | 1 | 1 | 17 |
| Observes negative consequences | 1 | 1 | 1 | 3 | 3 | 5 | 0 | 0 | 1 | 2 | 17 |
| Comorbidity | 1 | 1 | 1 | 0 | 0 | 6 | 2 | 1 | 2 | 1 | 15 |
